# Ancient DNA reveals genetic connections between early Di-Qiang and Han Chinese

**DOI:** 10.1186/s12862-017-1082-0

**Published:** 2017-12-04

**Authors:** Jiawei Li, Wen Zeng, Ye Zhang, Albert Min-Shan Ko, Chunxiang Li, Hong Zhu, Qiaomei Fu, Hui Zhou

**Affiliations:** 1 College of Life Science, Jilin University, Changchun, 130023 People’s Republic of China; 2 Ancient DNA Laboratory, Research Center for Chinese Frontier Archaeology, Jilin University, Changchun, 130012 People’s Republic of China; 3 Key Laboratory of Vertebrate Evolution and Human Origins of Chinese Academy of Sciences, IVPP, CAS, Beijing, 100044 People’s Republic of China

**Keywords:** Di-Qiang population, Ancient DNA, Mitochondrial DNA, Non-recombining region of the Y-chromosome, Han Chinese population

## Abstract

**Background:**

Ancient Di-Qiang people once resided in the Ganqing region of China, adjacent to the Central Plain area from where Han Chinese originated. While gene flow between the Di-Qiang and Han Chinese has been proposed, there is no evidence to support this view. Here we analyzed the human remains from an early Di-Qiang site (Mogou site dated ~4000 years old) and compared them to other ancient DNA across China, including an early Han-related site (Hengbei site dated ~3000 years old) to establish the underlying genetic relationship between the Di-Qiang and ancestors of Han Chinese.

**Results:**

We found Mogou mtDNA haplogroups were highly diverse, comprising 14 haplogroups: A, B, C, D (D*, D4, D5), F, G, M7, M8, M10, M13, M25, N*, N9a, and Z. In contrast, Mogou males were all Y-DNA haplogroup O3a2/P201; specifically one male was further assigned to O3a2c1a/M117 using targeted unique regions on the non-recombining region of the Y-chromosome. We compared Mogou to 7 other ancient and 38 modern Chinese groups, in a total of 1793 individuals, and found that Mogou shared close genetic distances with Taojiazhai (a more recent Di-Qiang population), Hengbei, and Northern Han. We modeled their interactions using Approximate Bayesian Computation, and support was given to a potential admixture of ~13-18% between the Mogou and Northern Han around 3300–3800 years ago.

**Conclusions:**

Mogou harbors the earliest genetically identifiable Di-Qiang, ancestral to the Taojiazhai, and up to ~33% paternal and ~70% of its maternal haplogroups could be found in present-day Northern Han Chinese.

**Electronic supplementary material:**

The online version of this article (10.1186/s12862-017-1082-0) contains supplementary material, which is available to authorized users.

## Background

The Huaxia is the earliest Chinese dynasty to emerge ~2000 BC along the Yellow River. This population grew from the Central Plain area and later became established as the Han Chinese during the Han Dynasty (206 BC to 220 AD). Throughout history, the Han Chinese continued to have complex interactions with surrounding ethnic minority groups in their vicinity [1, 2], whose details are being studied and debated by historians, archaeologists, anthropologists and geneticists.

One important pastoral agriculturist group that interacted with the Han Chinese from the west near the upper reaches of the Yellow River in the Gansu-Qinghai (or Ganqing) region is a historical group called the Di-Qiang. Around the middle Neolithic, as people (including ancestors of the Han) expanded away from the Central Plain due to improved agricultural practices [3], they encountered the Di-Qiang people, and both groups have occupied the Ganqing [4, 5]. A recent ancient DNA study goes further to suggest that a once Ganqing population, the Taojiazhai people, is related to the Di-Qiang, and even contributed genetically to the Han Chinese [6]. However, an issue with the Taojiazhai was that the archeological site dated to ~1700-1900 yr. BP, which occurred well within the time period of the Han dynasty, raising the possibility that some Taojiazhai individuals might have been admixed in Han Chinese.

**Fig. 1:**
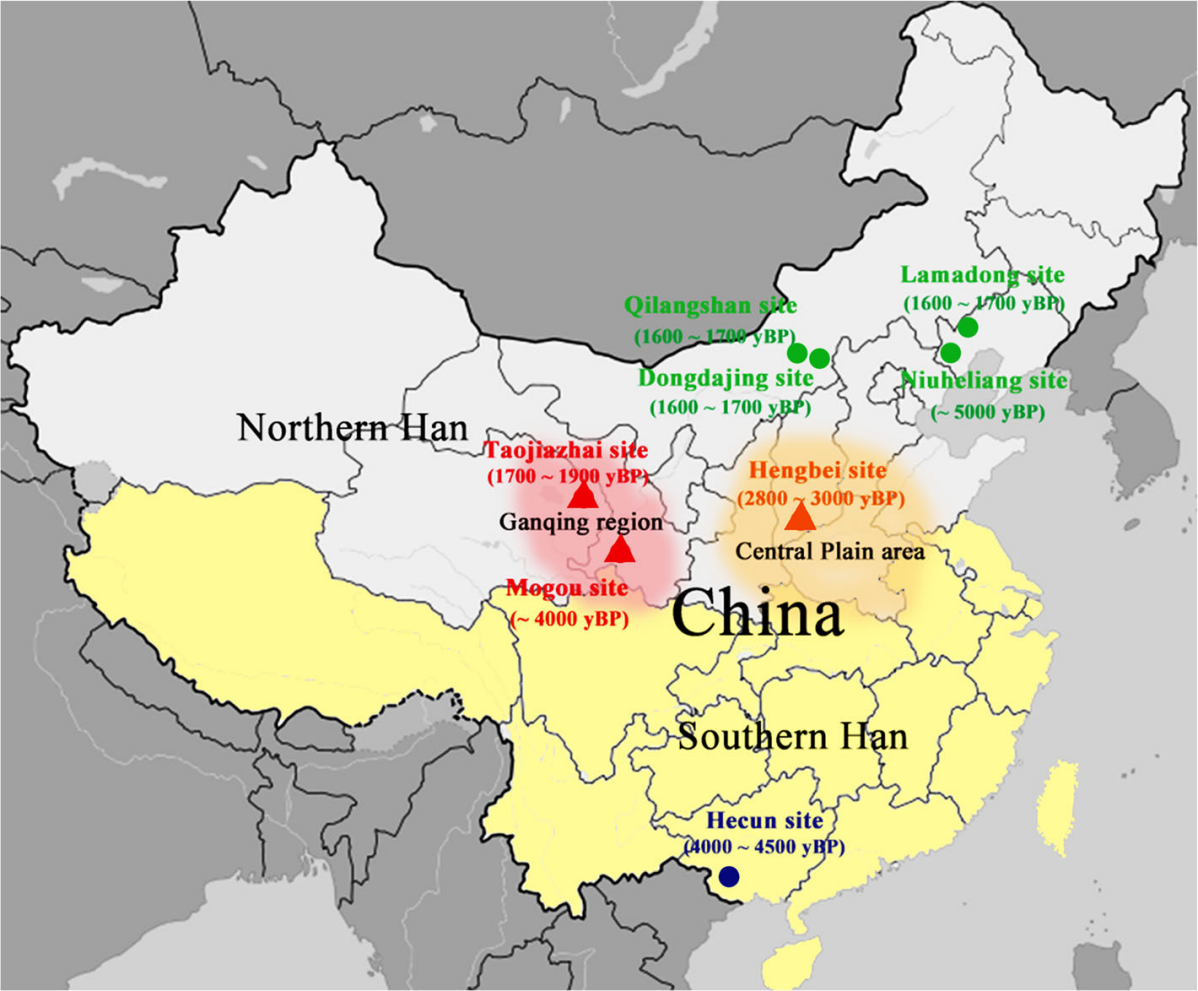
Geographic location and estimated age of ancient groups used in this study. Ganqing region (shaded red) overlies the middle and upper reaches of the Yellow River and is adjacent to the Central Plain area (shaded orange), where ancestors of the Han lived

In this study, we overcome this problem by investigation of the Mogou cemetery (Fig. 1), a considerably older Di-Qiang site in the Ganqing region that is enclosed by the Qinghai-Tibetan Plateau to the west and the Tengger Desert to the north [7]. The accelerator mass spectrometry (AMS) radiocarbon dating of the Tomb M633 human bone samples (slightly more recent than specimens collected for this study) yielded 3145 ± 45 ^14^C yr. BP and 3526–3336 cal. yr. BP after correction with Damon’s table [8]. Cultural artifacts, such as funerary pottery constructed of red clay with features found prominently in the Qijia culture, place this site in the late Neolithic to early Bronze Age (~3600 to 4200 yr. BP) [9] and associated with the Di-Qiang [10]. So the Mogou represents an early Di-Qiang predating the Han dynasty.

To clarify the genetic relationship between the ancient Di-Qiang and early Han Chinese, we analyzed the new Di-Qiang from Mogou, using hyper variable sequence I (HVS-I) and coding region of mtDNA and non-recombining region of Y-chromosome (NRY), and compared them to other ancient DNA and contemporary groups across China.

## Methods

### Population samples and Mogou specimens

A total of 1793 individuals were collected, belonging to 8 ancient (235 individuals) and 38 modern (1558 individuals) groups (Table 1). The Mogou site (34°69′N 103°86′°E) is located in the Tibetan Autonomous Prefecture of Gannan, Gansu Province, being at the geographical center of China and upstream of the Yellow river [7]. The high altitude (2209 to 3926 m) and a continental climate with an annual average temperature of 3.28 °C are favorable to ancient DNA preservation [11]. The Institute of Cultural and Historical Relics and Archaeology in Gansu Province excavated the cemetery, consisting of 26 graves, with permission from the State Administration of Cultural Heritage who has control over the archaeological excavations in China. Nearly every grave contained multiple individuals due to the complex structure of the tomb. In total, 60 ancient human remains were exhumed from the Mogou site for genetic analysis (see Additional file 1).

**Table 1:** List of populations used in this study

Ancient DNA population	*n*	Approx. age (yr BP)	Sampled location	References
Mogou	55	4000	Gansu	This study
Hecun	9	4500	Guangxi	Unpublished
Lamadong	17	1700	Liaoning	[38]
Hengbei	64	3000	Shanxi	[40]
Niuheliang	28	5000	Liaoning	[41]
Taojiazhai	29	1900	Qinghai	[6]
Dongdajing	17	1700	Inner Monglia	[39]
Qilangshan	16	1700	Inner Monglia	[37]
Modern population	*n*	Groups	Sampled location/ethnicity (*n*)	
Northern Han	267	5	Gansu (45), Qinghai (51), Shaanxi (53), Liaoning (51), Jiangsu (67)	[16, 17, 42, 43]
Northern minorities	192	4	Ewenki (47), Korean (48), Mongolian (49), Uygur (48)	[44, 46]
Southern Han	168	5	Fujian (54), Guangdong (29), Guangxi (26), Hunan (16), Yunnan (43)	[16, 17, 42, 43]
Miao-Yao	215	4	Hunan (102), Guangxi (73), Yunnan (40)	[48]
Tai-Kadai	397	11	Buyang (31), Caolan (30), Jiamao (27), Lingan (31), Mak (33), Mollao (29), Mulam (39), Pou (34), Sui (30), Then (30), Zhuang (83)	[46, 49]
Austro-Asiatic	84	3	Bugan (32), Palyu (30), Va (22)	[49]
Tibeto-Burman	235	6	Bai (90), Hani (33), Nakchu (30), Tibetan (35), Tujia (31), Yi (16)	[45–47]

### DNA extraction and laboratory environment

Prior to DNA extraction, the samples were cleaned using a series of treatments to remove the exogenous contaminants on the surface of the samples [12]. All of the operations were performed in separate rooms of an ancient DNA laboratory to strictly avoid any external contamination. Procedures were carried out independently of molecular biology experiments using present day DNA.

Before powdering, the bones and teeth surfaces were wiped down using cotton soaked in sodium hypochlorite solution. Bones and teeth were then soaked in a 5% sodium hypochlorite solution for 15 min, and carefully rinsed with 95% alcohol, and then UV-irradiated overnight. For dried bone material, a drilling machine was used to remove the top layer to avoid any remaining surface contaminants, and then powder was obtained for DNA extraction by drilling holes into the remainder of the bone. For the teeth, the dental calculus on the surfaces of the teeth was removed before drilling dental cervix to obtain cavitas pulpae powder. A turbid solution was then created, containing a mixture of about 200 mg of bone or tooth powder and 4.5 mL 0.5 mM EDTA (pH 8.0). This solution was stored at 4 °C for 24 h, upon which 80 μL of 100 mg/mL of proteinase K was added. The resulting solution was placed in the hybridization oven overnight at 56 °C. The precipitate was removed by centrifugation (3 min at 8000 rpm), and the clear supernatant extract was concentrated to 100μL using an ultrafiltration tube (Centurion® YM-10) at 8000 rpm centrifugation. DNA was extracted using the concentrated solution in accordance with the QIAamp® Purification Kit manual. Furthermore, DNA extraction was performed at least twice for each sample, and every five ancient samples had one blank control.

### Measures taken to ensure authenticity

To ensure the results are valid and reliable, we have kept in strict compliance with the rules indicated for extracting ancient DNA [13]. All laboratory personnel involved in the operation were female. Moreover, to obtain satisfactory results in ancient DNA research, the two guidelines were followed:

1. Pre-PCR and post-PCR protocols were carried out in two completely separate buildings. Experimenters were only allowed to move from the pre-PCR lab building to the post-PCR lab building each day, avoiding contamination from PCR products into the samples. The reverse was not allowed. The experimental areas including both the PCR room and the DNA extraction room have been equipped with Air Shower, which removes the dust, hair and other debris attached to clothes and reduces introduction of contaminants from laboratory personnel.
2. During the study period, we relocated our laboratory to a new campus, creating an opportunity to observe whether our results could be replicated in the new laboratory. Furthermore, different parts of the samples were randomly selected for replicate extraction and PCR amplification, in order to ensure the results are reproducible.

### Mitochondrial DNA amplification and haplogroup assignment

Due to high degradation of DNA from the ancient samples, it is difficult to amplify long DNA fragments. We thus designed two sets of primers (see Additional file 2) to amplify and sequence the mtDNA HVS-I region between positions 16,051 and 16,384. We also used both the Sanger sequencing method and the amplified product-length polymorphisms (APLP) method [14, 15], through the design of two or three sets of specific and corresponding primers (see Additional file 2).

The PCR amplification was carried out in a 12.5 μL reaction mixture containing 2 μL of template DNA, 1.5× reaction buffer (Takara, Japan), 2.5 mM MgCl_2_ (Promega, Germany), 0.25 mM dNTPs (Takara, Japan), 0.1 μM of each primer, 1 U of *ExTaq*®Hot Start Version DNA polymerase (Takara, Japan), 1 μL 20 mg/mL BSA, and RNase-Free Water (Takara, Japan). Cycling parameters were described as follows: initial denaturation at 94 °C for 5 min, followed by 34 cycles at 94 °C for 30s, 30s at 55 °C, elongation for 30s at 72 °C, with a final extension for 10 min at 72 °C and storage at 4 °C. Then, the PCR amplification products were examined by agarose gel electrophoresis. After the purification with the QIA quick Gel Extraction Kit (Qiagen, Germany), the amplification products were sequenced using the BigDye® Terminator V3.1 Cycle Sequencing kit (Applied Biosystems, USA). These sequences were analyzed, and an output file was generated from the ABI PRISM™310 automatic sequencer. In the end, the mtDNA haplogroups were called based on SNPs from the hypervariable and the coding regions, and the East Asian mtDNA classification tree [16, 17].

### Sex identification and Y-DNA haplogroup assignment

The sex of the ancient specimens was determined by PCR analysis of the X-Y Amelogenin Gene (AMG-PCR) [18]. The primers are listed in Additional file 2. The Y Chromosome SNPs M9, M214, M175, M122, M324, and P201 were typed for the detection of the following haplogroups: K, NO, O, O3, O3a, and O3a2 [19–22]. The Y-DNA haplogroups were called according to SNPs listed in ISOGG 2014 (https://isogg.org/).

### Non-recombining region of the Y chromosome (NRY) capture

We performed NRY capture of two Mogou males (MG18 and MG48). The DNA library was prepared with NEBNext® Ultra™ DNA Library Prep Kit for Illumina® in accordance to manufacturer’s instructions, which is similar to the Illumina TruSeq V2 protocol [23]. This library preparation will perform end repair with 5′ phosphorylation and dA-tailing, and make the damage-derived C-to-T in the 5′-endoverhang fragments to have the reverse complement nucleotides G-to-A substitutions at the 3′-end. After the ligation with NEBNext Adaptors (that includes hairpin loop with uracil), the uracils in the adaptor and DNA insert are then removed by USER (uracil-DNA-glycosylase (UDG) and endonuclease VIII), which would cause a small residual signal of C-to-T substitutions to be detected at the 5′ (~1.8%) and no influence to the G-to-A substitutions at 3′ terminal positions (for MG18 ~11% and for MG48 ~16%) [23]. However, within a CpG context, because the majority of cytosines are methylated invertebrate genomes, which when deaminated, leaves thymine instead of uracil, the deaminated cytosines in the majority of cases are not removed in this CpG part even with the USER treatment (CpG part: C-to-T substitutions for MG18 ~11% and for MG48 ~15%, see Additional file 3).

Next, the 7.18 Mb targeted unique regions on the NRY chromosome was used to design the array. We used a similar experimental method of the one described by Fu et al. [24], to do the in-solution hybridization enrichment for the libraries. We then focused on the reads passing Illumina quality control that had the expected index combinations for these libraries. We sequenced the libraries on the HiSeq X-Ten platform. We restricted analysis to pairs of reads that had at least 11 base pairs of overlap, merged the reads, and then mapped the merged sequences to the human genome reference hg19 using BWA. We removed duplicated molecules prior to analysis to reduce the influence of mapping errors. We restricted our analysis to unique regions in the genome, using Tandem Repeat Finder (for hg19) and mapability tracks (map 35-50%). Details of sequencing coverage on NRY are shown in Table 2. The fragments size distribution of two Mogou male specimens show short length fragments, which are typical for ancient DNA (see Additional file 4).

**Table 2:** Sequencing metrics for two libraries of NRY capture

Library	Total fragments (length ≥ 35 bp)	Aligned fragments (length ≥ 35 bp & map quality > = 30)	Fragments on target (length ≥ 35 bp & map quality > = 30)	Fragments on target after duplicate removal (length ≥ 35 bp)	Average duplicates	Coverage
MG18	21,590,365	4,455,311	4,095,184	44,977	91	0.59
MG48	23,458,692	13,692,019	10,262,330	752,478	14	8.51

### Genetic analysis

Chromas 2.4.1 and Sequencher 5.2.3 were used for sequence assembly and to check sequence alignment. Genetic distances (based on Fst [25]) between populations are shown using Multidimensional Scaling (MDS), and Analysis of Molecular Variance (AMOVA) were calculated in Arlequin (v3.5.1.2) [26]. The mtDNA haplogroup frequencies were shown using Principal Component Analysis (PCA). Temporal networks or TempNet [27], which shows networks stacked in three dimensions (3-D), was used to explore the continuity of haplotypes across time. The phylogeny of Y-DNA haplogroup O was inferred using Figtree in the BEAST program [28] and tip dating of ancient DNA [29]. Demographic histories were simulated using Fastsimcoal [30] and parameter distributions inferred by Approximate Bayesian Computation (ABC) [31].

## Results

A total of 55 of 60 samples from the Mogou site (Additional file 1) were successfully replicated, and verified to be different from the mtDNA of laboratory personnel (see Additional file 5). All sequences were submitted to GenBank under the accession numbers KX085423-KX085477.

### Mitochondrial DNA analysis

The organic preservation was relatively high for Mogou, most likely related to its high elevation and low temperature. For example, from the captured MG48 library, we obtained 97-fold coverage for the mtDNA genome. The contamination of MG48 was 0.048% (95% C.I. 0.545%-0.008%) based on the match rate to the mtDNA consensus by running the contamination estimator ContamMix [24]. We genotyped 55 samples for mtDNA HVS-I and nt1040 0 T/C (for the M/N type), and further SNP loci detection was carried out on the coding region to ensure that haplogroup was correctly called based on the results of the HVS-I motif. We found a total of 46 haplotypes (Table 3) with certain haplotypes shared by two or more individuals buried in the same grave, suggesting a matrilineal kinship among some individuals. The haplotypes were analyzed using the correction criterion developed by Alzualde et al. [32] to control for the reduction in the genetic diversity due to kinship [33]. Table 3 shows these haplotypes could be assigned to 14 mtDNA haplogroups: A, B, C, D (D*, D4, and D5), F, G, M7, M8, M10, M13, M25, N*, N9a, and Z. Additional file 6 shows the more frequent Mogou haplogroups were D (34.78%), C (10.87%), A (8.70%), and F (8.70%), while the M8, M13, M25, and N* have only one individual each. Most of these haplogroups occur among East Asians [16] with M25 found in South Asians [34].

**Table 3:** mtDNA nucleotide changes in 55 Mogou samples

Haplotype	Specimen	Haplogroup	Coding region SNPs	Mutations in mitochondrial HVS-I (160001+)
ht1	MG28	A	10,400, 663	086-223-290-319-362
ht2	MG20	A	10,400, 663	093-129-223-284-290-319-362
ht3	MG23	A	10,400, 663	051-129-182C-183C-189-290-319-362
ht4	MG24	A	10,400, 663	223-290-311-319-362
ht5	MG8	B	9 bp deletion	111-140-183C-189-234-243
ht6	MG57			
ht7	MG21	C	10,400, 14,318	223-298-327
ht8	MG37			
ht9	MG36	C	10,400, 14,318	093-129-223-298-327
ht10	MG41	C	10,400, 14,318	093-129-188-223-298-327
ht11	MG50,MG51, MG54	C	10,400, 14,318	129-192-223-298-327
ht12	MG38	D*	10,400, 5178	129-223-362
ht13	MG33	D*	10,400, 5178	151-223-290-362
ht14	MG11	D4	10,400, 5178, 3010	223-362
ht15	MG22			
ht16	MG47, MG52, MG53			
ht17	MG3, MG5, MG6	D4	10,400, 5178, 3010	223-274-362
ht18	MG45			
ht19	MG48	D4	10,400, 5178, 3010	129-223-274-362
ht20	MG4	D4	10,400, 5178, 3010	223-311-362
ht21	MG40	D4	10,400, 5178, 3010	223-343-362
ht22	MG19	D4	10,400, 5178, 3010	223-292-311-328-362
ht23	MG10	D5	10,400, 5178,10,397	126-182C-183C-189-223-362
ht24	MG55	D5	10,400, 5178,10,397	129-164-172-182C-183C-189-223-266-362
ht25	MG58			
ht26	MG18	D5	10,400, 5178,10,397	092-164-172-182C-183C-189-223-266-362
ht27	MG32			
ht28	MG35	F	3970	189-304
ht29	MG42			
ht30	MG44	F	3970	183C-189-304
ht31	MG31	F	3970	183C-189-232A-249-304
ht32	MG59	G	10,400, 4833	129-223-261-278-311-362
ht33	MG43	G	10,400, 4833	086-153-223-278-362
ht34	MG25	M7	10,400, 6455	223-295-362
ht35	MG27	M7	10,400, 6455	223-294-295-362
ht36	MG14	M8	10,400, 15,487 T	184-223-298-319
ht37	MG9	M10	10,400, 10,646	223-311
ht38	MG29, MG30			
ht39	MG49	M10	10,400, 10,646	093-129-193-223-311-357
ht40	MG39	M13	10,400, 6023	145-188-189-192-223-311-381
ht41	MG60	M25		223-304
ht42	MG2	N*	10,398	223-243-256
ht43	MG7	N9a	5417	114-223-261
ht44	MG13	N9a	5417	223-257A
ht45	MG15, MG16, MG17	Z	10,400, 15,784	185-223-260-298
ht46	MG12	Z	10,400, 15,784	185-223-259-260-298

AMOVA was used to test how different classifications would affect the variance among groups (Additional file 7). We found geography explained the most variance among groups. Compared to the variance given when all groups were independent (variance among groups = 2.01%), the highest variance (variance among groups = 1.64%) was observed when two geographic groups were classified, i.e. Northern China (Mogou, Hengbei, Taojiazhai, Northern Han and Northern minorities, Tibeto-Burman) and Southern China (Southern Han and Southern minorities). The Tibeto-Burman was better grouped with Northern China than independently (variance among groups = 1.33%) or with Southern China (variance among groups = 1.29%). The ancient groups (Mogou, Hengbei, Taojiazhai) associated more with Northern Han (variance among groups = 1.36%) than with Northern minorities (variance among groups = 1.09%).

### Y-chromosome analysis

Of the 55 samples, 15 males and 17 females were identified using molecular biology techniques. Remaining 23 samples were indeterminate for sex after testing the AMG-PCR product. Only six amplified Y-SNP products could be successfully replicated. Table 4 shows all six Mogou males belonged to Y-DNA haplogroup O3a2/P201. The two male specimens (MG18 and MG48) selected for capture 7.18 Mb of the NRY chromosome, after retaining positions with coverage at least 3-fold, their Y-DNA haplogroups were identified to be O3a2c and O3a2c1a/M117, respectively. The MG48 was further analyzed since a higher 8.51-fold coverage was better to build the consensus.

**Table 4:** Y-DNA haplogroup-defining SNPs of Mogou males

Specimen	C	F	K	NO	N	O	O3	O3a	O3a2	Haplogroup
	M216	M89	M9	M214	M231	M175	M122	M324	P201	
	C → T	C → T	C → G	T → C	G → A	−5 bp	T → C	C → G	T → C	
MG3	C	T	G	C	G	−5 bp	C	G	C	O3a2
MG9	C	T	G	C	G	−5 bp	C	G	C	O3a2
MG18	C	T	G	C	G	−5 bp	C	G	C	O3a2
MG44	C	T	G	C	G	−5 bp	C	G	C	O3a2
MG48	C	T	G	C	G	−5 bp	C	G	C	O3a2
MG53	C	T	G	C	G	−5 bp	C	G	C	O3a2

**Fig. 2:**
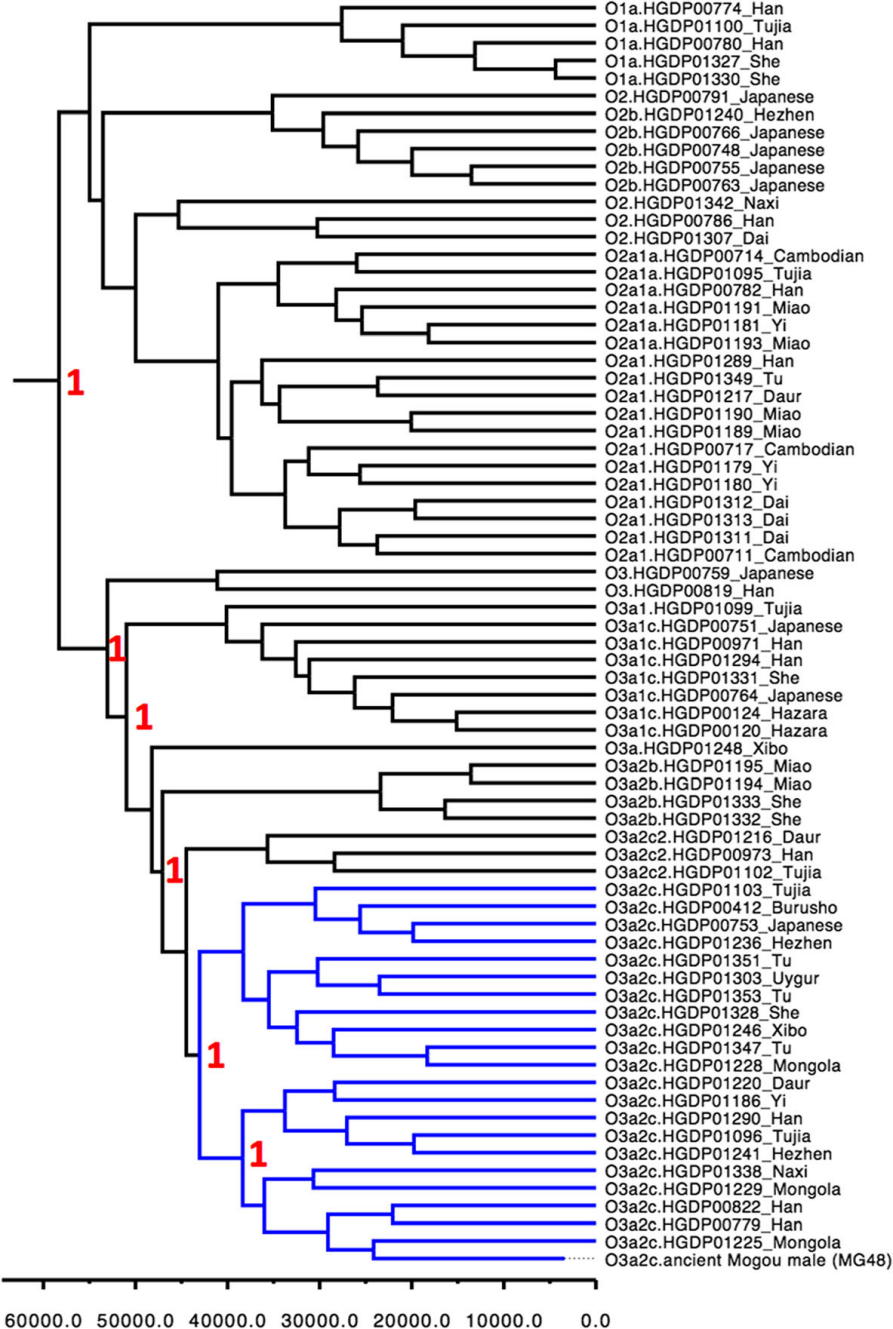
Mogou male (MG48) was grouped under O3a2c on Y-DNA haplogroup O lineage using BEAST. O3a2c branches (blue); X-axis denotes time in yr. BP, and posterior values shown in red

We aligned MG48 with the published 71 HGDP East Asian individuals with O haplogroup [35] to verify that it could be properly placed within the haplogroup O lineage. The consensus length, between MG48 (retaining positions with coverage of at least 3-fold) and HGDP Y-chromosome dataset, was 381,473 bp. Figure 2 shows that all 72 sequences could be confidently assigned to Y-DNA haplogroup O1, O2, O3 using 31 ISOGG defining SNPs, and that the posteriors leading up to the O3a2c clade that the MG48 falls under were 1.0, thus ensuring that its position was highly resolved. The inferred Y-DNA substitution rate of 7.76 × 10^−10^ (95% CI 3.89 × 10^−10^ to 1.13 × 10^−9^) per site per year remained consistent with other ancient DNA studies [36].

### MDS of mtDNA genetic distances (Fst)

**Fig. 3:**
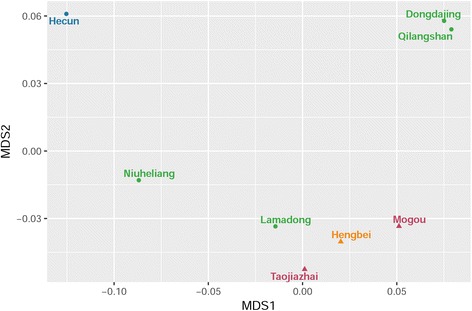
MDS plot of genetic distance Fst between 8 ancient groups

**Fig. 4:**
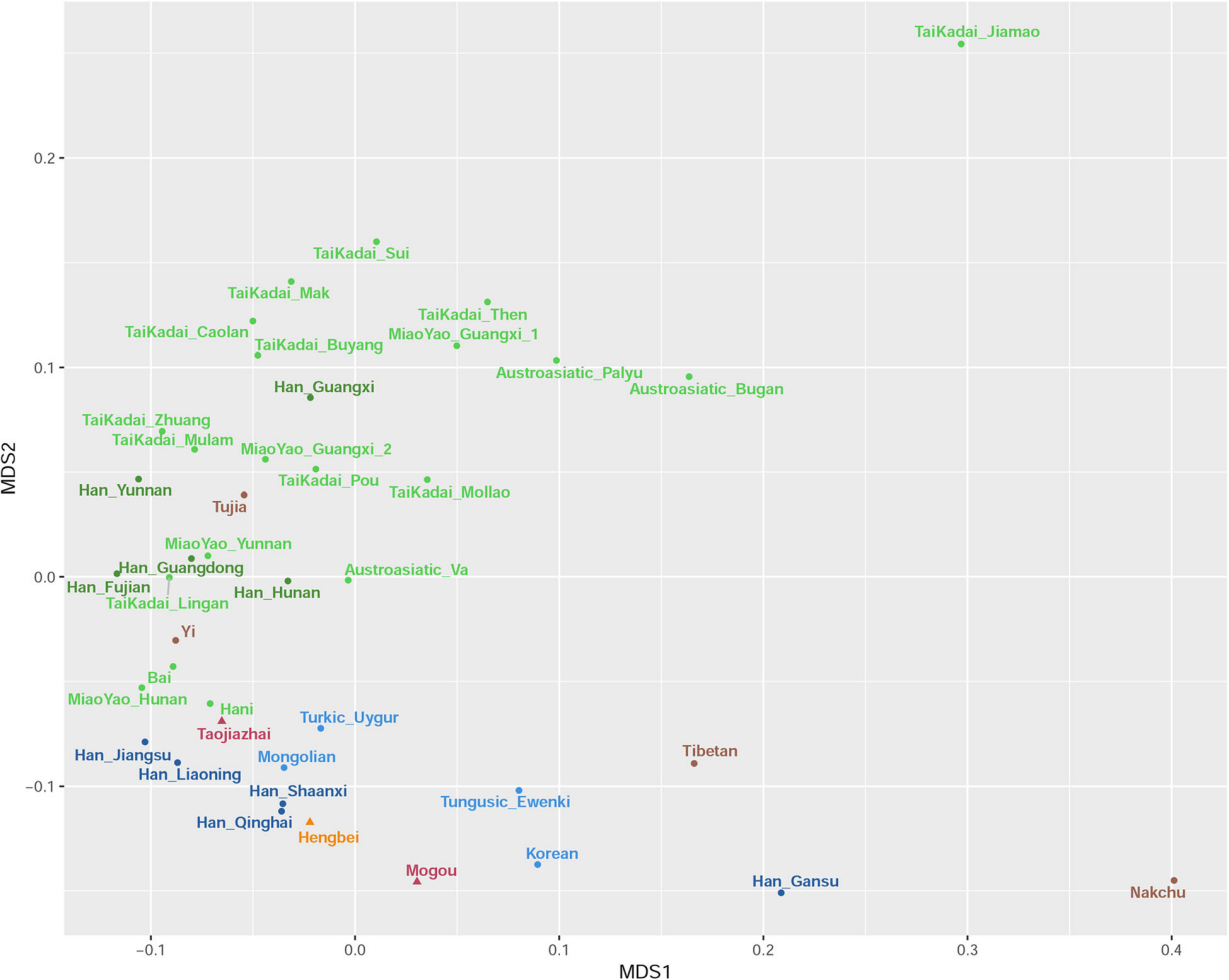
MDS plot of genetic distance Fst between 3 ancient and 38 modern Chinese groups

We calculated the genetic distance Fst and permutated *P*-values of the ancient [6, 37–41] and modern groups [16, 17, 42–49] based on mtDNA sequences. Additional file 8 shows that genetic distances were not significant between Mogou and Hengbei (Fst = 0.003), Taojiazhai (Fst = 0.005), Northern Han (Fst = 0.0004), Northern minorities (Fst = 0.005) then followed by Dongdajing (Fst = 0.01), Qilangshan (Fst = 0.02), and Lamadong (Fst = 0.02). However, there were significant genetic distances between Mogou and Hecun (Fst = 0.08), Niuheliang (Fst = 0.05), Southern Han (Fst = 0.03), Miao-Yao (Fst = 0.03), Tai-Kadai (Fst = 0.05), Austro-Asiatic (Fst = 0.04), and Tibeto-Burman (Fst = 0.01). Figure 3 shows MDS plot (MDS stress = 0.001) of a comparison among ancient samples, where Northern and Southern China divided in the first dimension, but Mogou, Hengbei and Taojiazhai clustered together. Figure 4 shows MDS plot (stress = 0.09) of a comparison between ancient and modern populations, where Mogou associated with the Northern minority and Southern minority (e.g. Austro-Asiatic) in the first dimension, and with the Hengbei and Northern Han (e.g. Shaanxi, Qinghai, Gansu) in second dimension. Hengbei and Taojiazhai both associated with the Northern Han (e.g. Han Shaanxi, Qinghai), and differed from Mogou in their increased associations with Southern minorities.

### PCA of mtDNA haplogroup frequencies

**Fig. 5:**
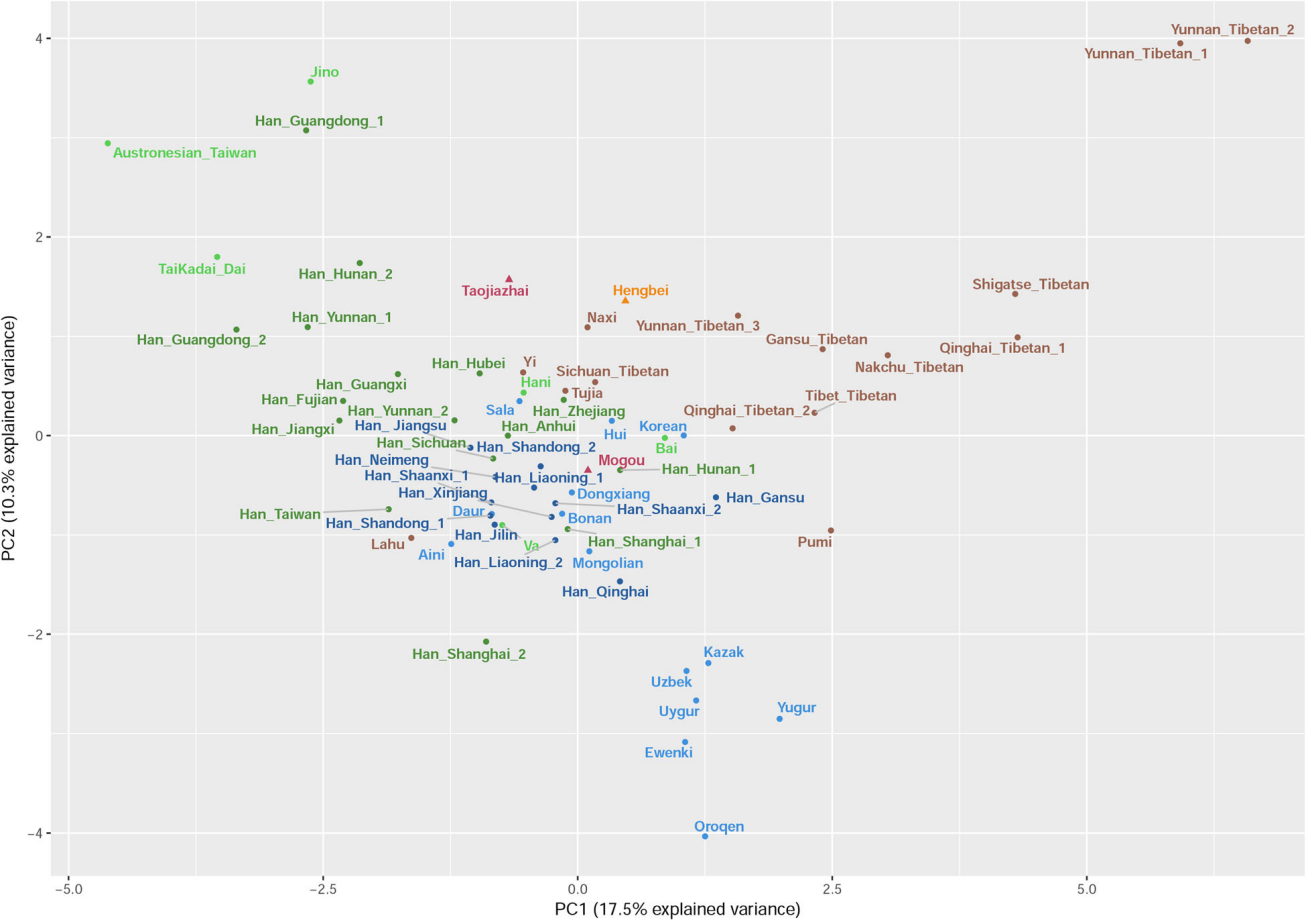
PCA plot of mtDNA haplogroup frequencies of 3 ancient and modern Chinese groups

Figure 5 shows Mogou is located at the center of PCA among the Chinese and Tibetans. Entering from bottom right are the Northern Han [16, 17, 42, 43] and Northern minorities [44, 46]. From the top left are Southern minorities [46, 48, 49] then the Southern Han [16, 17, 42, 43]. The Tibeto-Burman speakers [45–47] enters from the top right of this cluster (for details on mtDNA haplogroup frequencies, see Additional file 9).

### Temporal network analysis

**Fig. 6:**
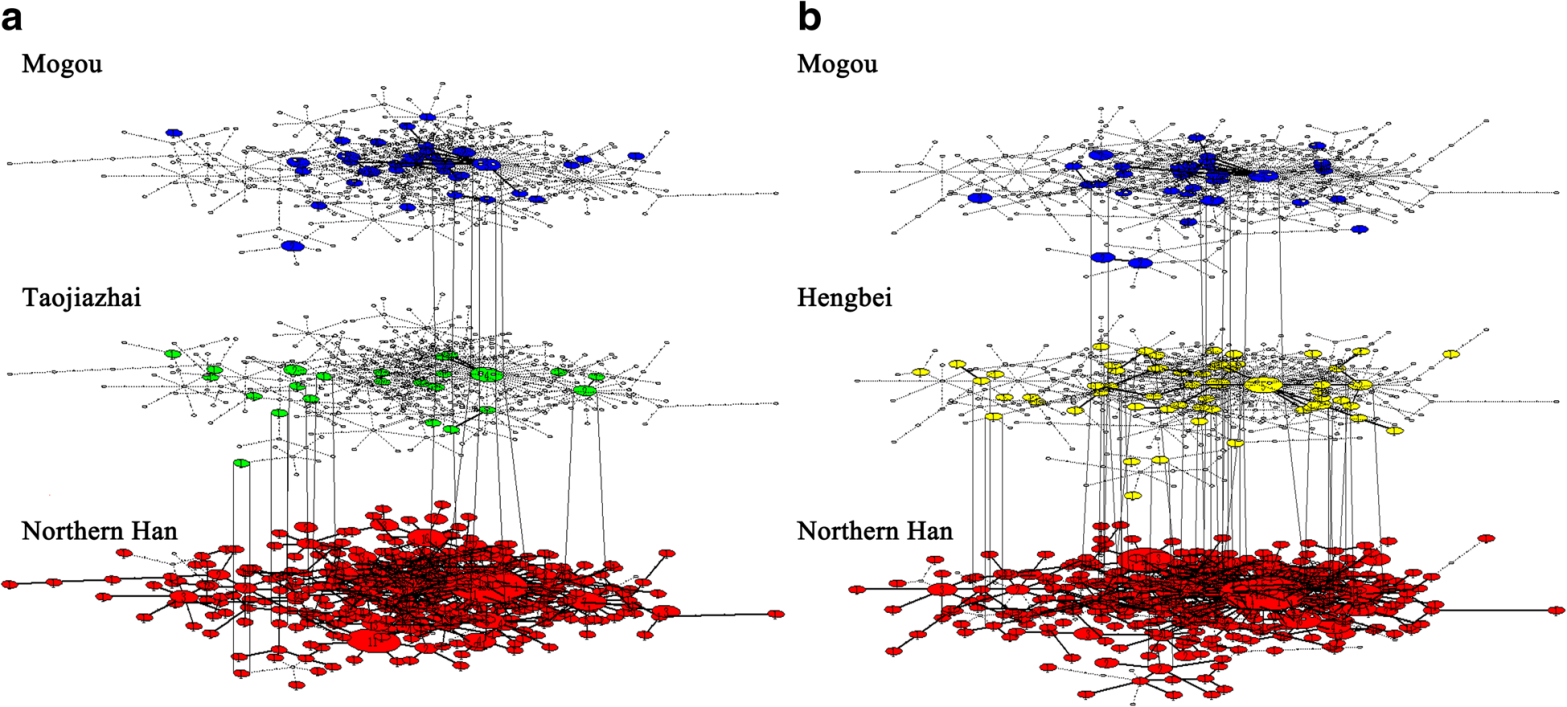
Temporal network of haplotype distribution across time. **a** Haplotype sharing across 46 Mogou individuals, 29 Taojiazhai individuals, and 521 Northern Han; **b** Haplotype sharing across 46 Mogou individuals, 64 Hengbei individuals, and 521 Northern Han

To identify whether Di-Qiang did have an influence on Han Chinese, we investigated the temporal network of Mogou, Taojiazhai, and Northern Han. Figure 6a shows haplotypes between Mogou and Taojiazhai were contiguous, and some sharing with Northern Han. Figure 6b shows that Mogou and Hengbei shared relatively more haplotypes with Northern Han compared to Taojiazhai.

### Approximate Bayesian computation (ABC) simulations

**Fig. 7:**
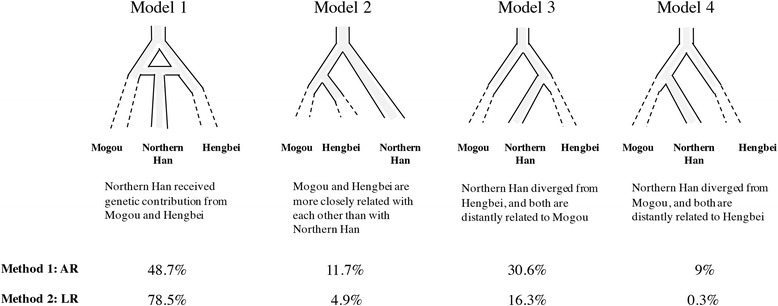
Probability of model 1 to 4 occurrence. Each model is followed by a brief description about their demographic history. Mogou and Hengbei are serial sampled at ~4000 and ~3000 yr. BP, respectively, and dashes indicate the uncertainty in whether they have direct modern descendants. In contrast Northern Han has solid line to the present-day. The probability (0-100%) of each model occurrence is assigned using AR (acceptance-rejection) and LR (logistic regression; details see main text)

To understand the genetic relationship between Mogou, Hengbei, and Northern Han, we proposed four models (Model 1–4; Fig. 7) that described the possible demographic history that occurred among them. After performing 1 million simulations for each model, the probability of model occurrence was assessed by two methods: acceptance-rejection (AR) [50] and weighted-multinomial logistic regression (LR) [31]. The quality of simulations was evaluated by R^2^, coverage, etc. compared to 1000 pseudo-observed, as described elsewhere [51]. The best supported model was Model 1 (49-79%) followed by Model 3 (16-31%; Fig. 7). We found the reason for the similarity between Model 1 and 3 was because Model 1 described Mogou contributed relatively few genes averaging at 15% (95% CI: 13-18%) into Northern Han around 3500 years ago (95% CI: 3301–3809; details in Table 5), which could approximate Model 3 that explained Northern Han is closer to Hengbei. The overall simulation quality was good, with the type I error (misclassified true models based on 1000 resamplings) of the four models being low ~18%. Every parameter were estimated with high coverage and R^2^ > 10% indicating that they were reliably estimated [51], and there were noticeable improvements (on average ~12-fold; Additional file 10) to the posteriors of summary statistics used.

**Table 5:** Parameter estimates of best supported Model 1

Population	Prior			Distribution	Mean	Mode	95% HPD		Pseudo-observed				
									R^2^	Bias	RMSE	Coverage	Factor 2
Mogou	1	–	100,000	Uniform	799	824	699	887	0.45	0.18	0.33	85	0.94
Hengbei	1	–	100,000	Uniform	1489	1559	1356	1759	0.84	0	0.11	90	1
Northern Han	1	–	100,000	Uniform	657	600	596	826	0.9	−0.1	0.26	90	1
T1	1	–	20,000	Uniform	5472	5661	5253	6389	0.76	−0.02	0.16	70	1
T2	1	–	20,000	Uniform	3505	3703	3301	3809	0.51	−0.02	0.15	80	1
a	0.01	–	1	Uniform	0.146	0.143	0.127	0.183	0.44	0.94	1.12	79	0.7

Mogou, Hengbei, Northern Han are effective haploids

T1, Divergence time between Mogou and Hengbei (yr BP)

T2, admixture time between Mogou and Hengbei (yr BP)

a, proportion of admixture from Mogou into Northern Han

μ, mtDNA (control region) substitution rate 9.883 × 10^−8^ per site per year were used according to ancient DNA-calibrated rates [52]

## Discussion

In this study, we found that Mogou, being situated at the geographic center of China, also lay at intersection of Northern and Southern Chinese and Tibetans in terms of haplogroup frequencies, suggesting it plays an important role in the formation of early cultures along the Yellow River. We argue that it possibly has a northern origin, since more than 90% of its maternal haplogroups (A, B, C, D, F, G, M7, M8, M10, N9a, and Z) matches with those typically found among ancient groups across Northern China. In particular, the most frequent haplogroup D in Mogou (34.78%) is consistent with other ancient northern groups (Qilangshan 43.75%; Dondajing 41.17%; Niuheliang 28.57%; Taojiazhai 27.59%; Hengbei 23.44%; Lamadong 17.6%) and less frequent in ancient southern group (Hecun 9.09%, unpublished). Genetic distance also shows that Mogou is closely related to two northern ancient groups (Hengbei and Taojiazhai). AMOVA further supports the grouping of these ancient DNAs alongside Tibetans and Northern Han and Northern minorities in explaining the highest variance among groups (1.64%; *P*-value <0.01).

The closest ancient relative to Mogou in our dataset was the Hengbei people (genetic distance Fst = 0.003), a ~3000 yr. BP population from the Central Plain region. Mogou and Hengbei shared about 33% Y-DNA haplogroup O3a/M324, as well as several maternal haplogroups (D, A, F, M10) and haplotypes (No. ht7, ht8, ht14, ht15, ht16, ht37, ht38, ht45). Because the temporal network showed a continuity of haplotypes across Mogou, Hengbei, and Northern Han, we investigated this further by constructing 4 model scenarios on how their relationship might have occurred. A higher model probability was assigned to a history where the Northern Han received ~13-18% maternal genes from Mogou around 3300–3800 years ago (Table 5) predating the formation of the Han.

The second closest ancient relative to Mogou was the Taojiazhai (genetic distance Fst = 0.005). All Mogou and Taojiazhai males shared 100% Y-DNA haplogroup O3a/M324, and many maternal haplogroups (D4, M10, F, Z) and haplotypes (No.ht14, ht15, ht16, ht28, ht29, ht37, ht38, and ht45). Few haplotypes were carried across to the Northern Han on the temporal network. However, the Taojiazhai appeared to differ from the Mogou in its increased association with the modern Southern Chinese in terms of haplogroup frequencies and Fst genetic distance.

The closest modern relative to Mogou was the Northern Han (genetic distance Fst = 0.002) and the Northern minorities (Fst = 0.005), and then more distantly by the Southern Han (Fst = 0.03) and Southern minorities (Fst = 0.03-0.05). Generally, the Y-DNA haplogroup O3a2/P201 from Mogou males is a common subtype of the O3a/M324 branch, which occurs at a high frequency in extant Han Chinese (43.37%) [43]. One Mogou male (MG48) was further identified as O3a2c1a/M117, which is a subclade of O3a2c1-M134 that is commonly found in Sino-Tibetan speakers and neighboring countries (e.g. Nepal, Bhutan, and Korea), but varies greatly in frequency among Miaoyao speakers. Furthermore, MG48 clustered with Han and Tibeto-Burman (e.g. Naxi, Yi, and Tujia) as opposed to southern groups (e.g. Dai, Miao) on the HGDP Y-DNA haplogroup O lineage (Fig. 2).

Finally, the present-day Tibeto-Burman speakers were also close to Mogou (genetic distance Fst = 0.01) than the Southern Han or Southern minorities. This was in agreement with historical records about the migration of ancient Di-Qiang people in the past. Some spread eastward, scattering in the middle reaches of the Yellow River, while others migrated southwest to form the Tubo, who are the ancestors of modern Tibetans, as well as contributing to the Southwestern minorities through integrating with the local population [5, 10].

## Conclusion

We identified Mogou to be the earliest ~4000 yr. BP Di-Qiang population, and genetically related to Taojiazhai in sharing up to 100% paternal (O3a) and ~60% maternal (D4, M10, F, Z) haplogroups. Among the alternative models considered, simulations demonstrated that Mogou and Hengbei once contributed genes into the early Northern Han. Thus, Mogou is also similar with the Northern Han in sharing up to ~33% paternal (O3a) and ~70% maternal (D, A, F, M10) haplogroups. We deduced that some Di-Qiang people had merged into the ancestral Han population. As societies developed, the communication and blending of different regions and cultures continued to be strengthened.

## Additional files

Additional file 1: Sampling information of Mogou site. (XLSX 13 kb)

Additional file 2: Primers used in this study. (XLSX 11 kb)

Additional file 3: Damage pattern of two Mogou male specimens MG18 (a) and MG48 (b). Only sequences of at least 35 bp that aligned to the human genome with a map quality of at least 30 were considered for this figure. Substitution frequencies are shown both for CpG and non-CpG context. (PDF 806 kb)

Additional file 4: Fragment size distribution of two Mogou male specimens MG18 (a) and MG48 (b). Only 8% of sequences merged from overlapping paired-end reads were considered for this figure. (PDF 178 kb)

Additional file 5: Variable sites of mitochondrial HVS-I sequences and Y chromosome haplogroups of researchers. (XLSX 11 kb)

Additional file 6: mtDNA haplogroup frequencies of 55 Mogou samples. (PDF 54 kb)

Additional file 7: Result of AMOVA variance explained by different groupings of populations according to geography and language. (XLSX 17 kb)

Additional file 8: Population pairwise genetic distance Fst. (XLSX 12 kb)

Additional file 9: Haplogroup frequencies of Mogou, Hengbei, Taojiazhai, and modern Chinese groups. (XLSX 26 kb)

Additional file 10: Bayesian posterior output of Model 1. (XLSX 12 kb)

## Abbreviations

3-D: Three dimensions
ABC: Approximate Bayesian Computation
AMG-PCR: PCR analysis of the X-Y Amelogenin Gene
APLP: Amplified product-length polymorphisms
AR: Acceptance-rejection
HVS-I: Hyper variable sequence I
LR: Weighted-multinomial logistic regression
mtDNA: Mitochondrial DNA
NRY: Non-recombining region of Y-chromosome
PCA: Principal Component Analysis
PCR: Polymerase chain reaction
SNP: Single nucleotide polymorphism
