# Supplementary figures and images for: Ancient DNA reveals genetic connections between early Di-Qiang and Han Chinese
Source: BMC Evol Biol. 2017 Dec 4;17:239. doi: 10.1186/s12862-017-1082-0 (PMC5716020; doi:10.1186/s12862-017-1082-0)

(a)

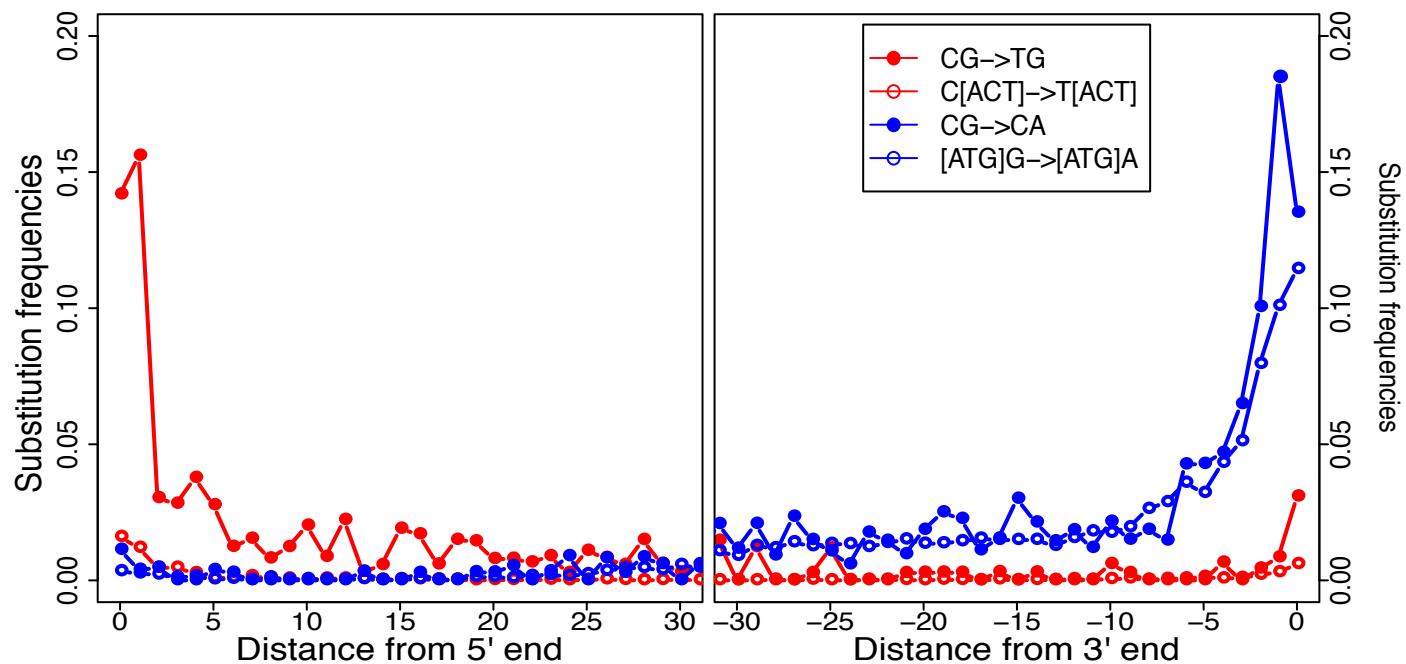

(b)

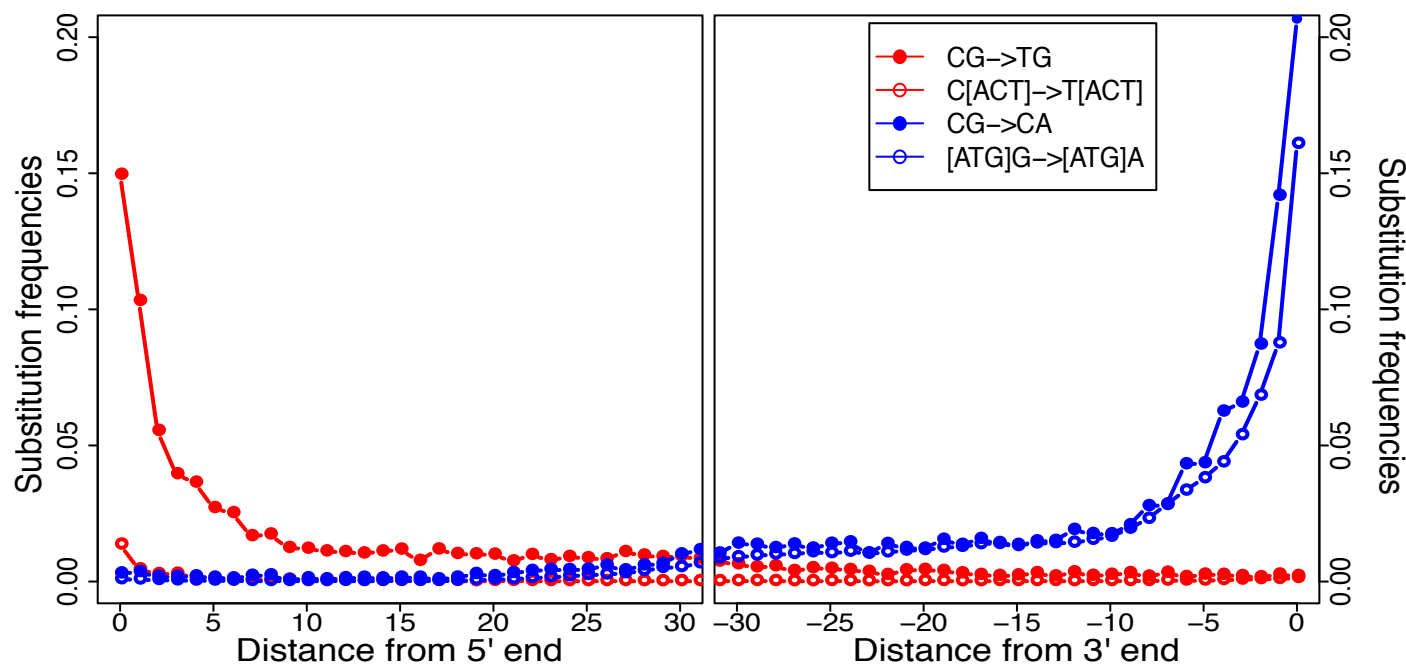

c)

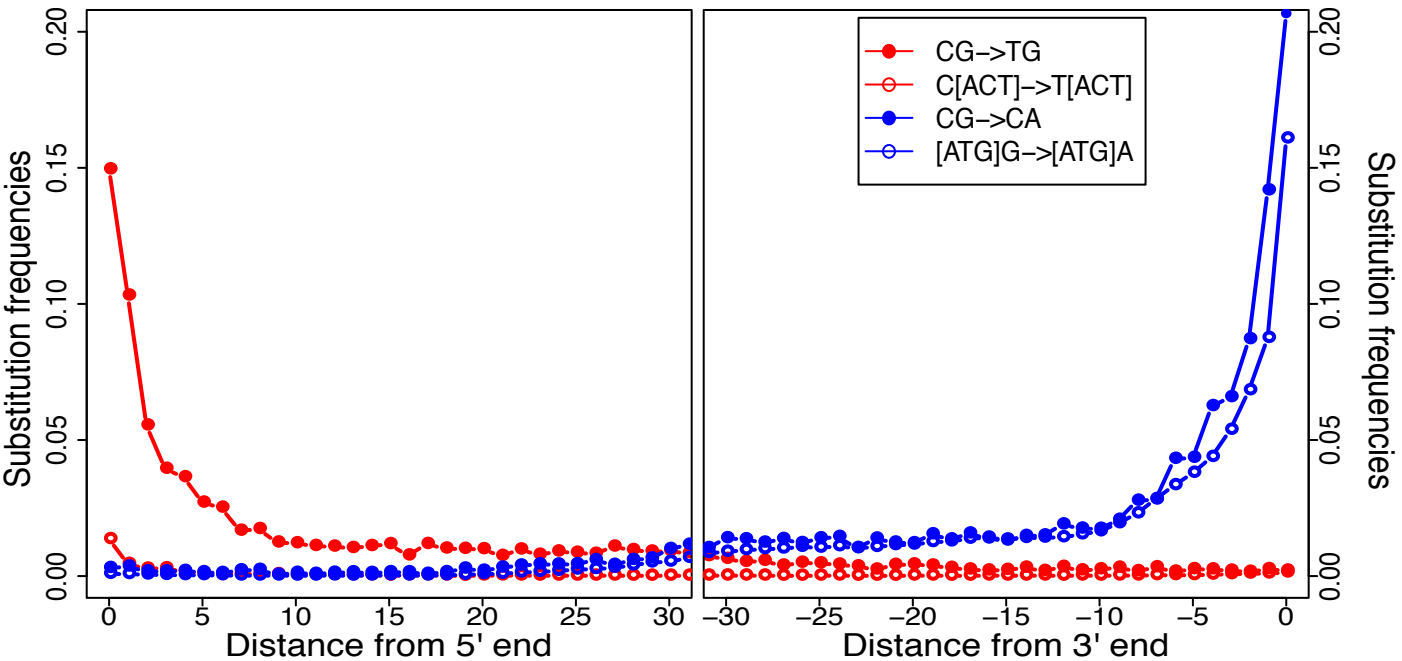

Supplement: Supplementary file 3 — Damage pattern of two Mogou male specimens MG18 (a) and MG48 (b). Only sequences of at least 35 bp that aligned to the human genome with a map quality of at least 30 were considered for this figure. Substitution frequencies are shown both for CpG and non-CpG context. (PDF 806 kb) [file 12862_2017_1082_MOESM3_ESM.pdf]

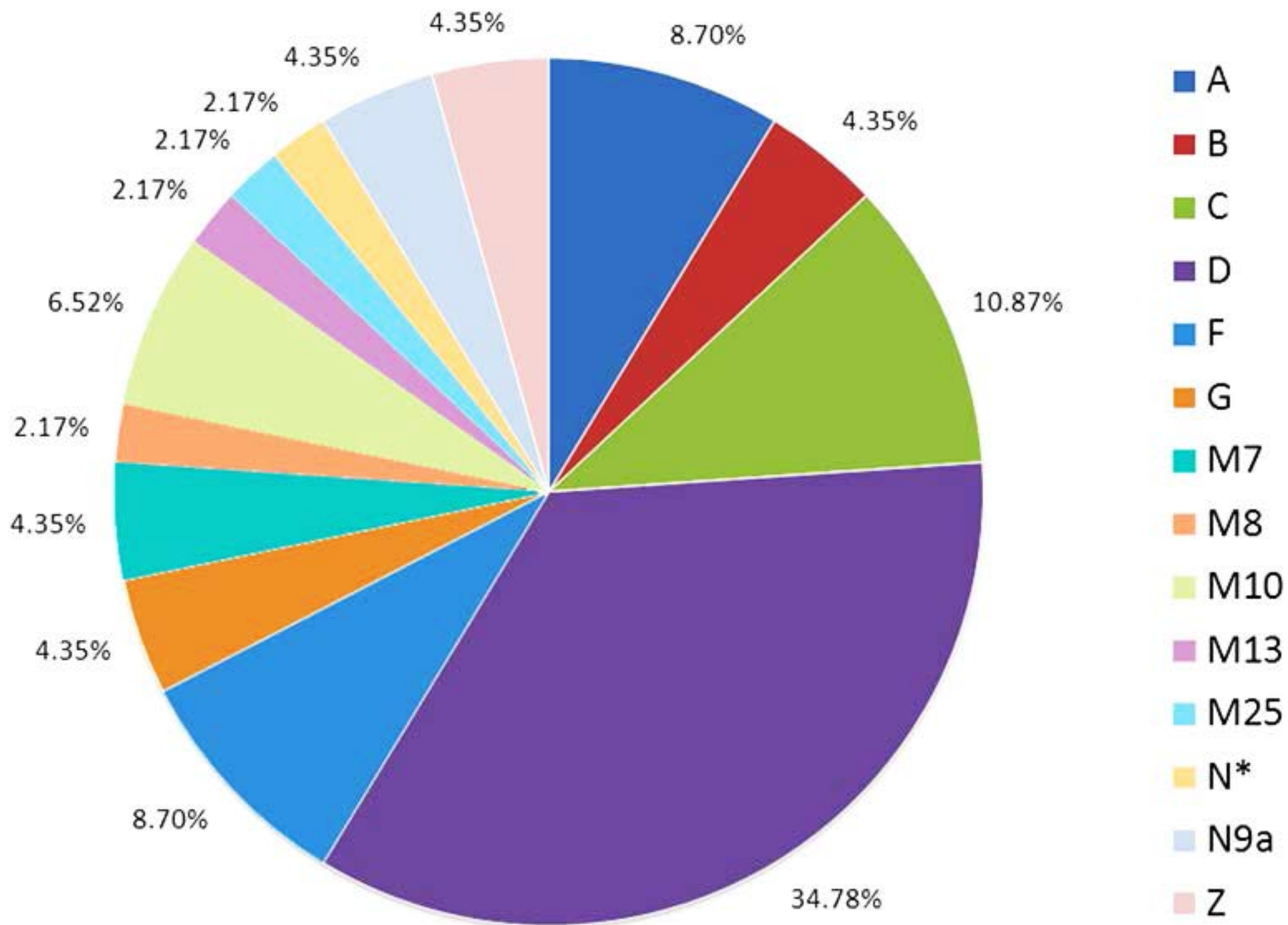

Supplement: Supplementary file 6 — mtDNA haplogroup frequencies of 55 Mogou samples. (PDF 54 kb) [file 12862_2017_1082_MOESM6_ESM.pdf]
