# Supplementary material for: Ancient DNA reveals genetic connections between early Di-Qiang and Han Chinese
Source: BMC Evol Biol. 2017 Dec 4;17:239. doi: 10.1186/s12862-017-1082-0 (PMC5716020; doi:10.1186/s12862-017-1082-0)
Supplement: Supplementary file 4 — Fragment size distribution of two Mogou male specimens MG18 (a) and MG48 (b). Only 8% of sequences merged from overlapping paired-end reads were considered for this figure. (PDF 178 kb) [file 12862_2017_1082_MOESM4_ESM.pdf]

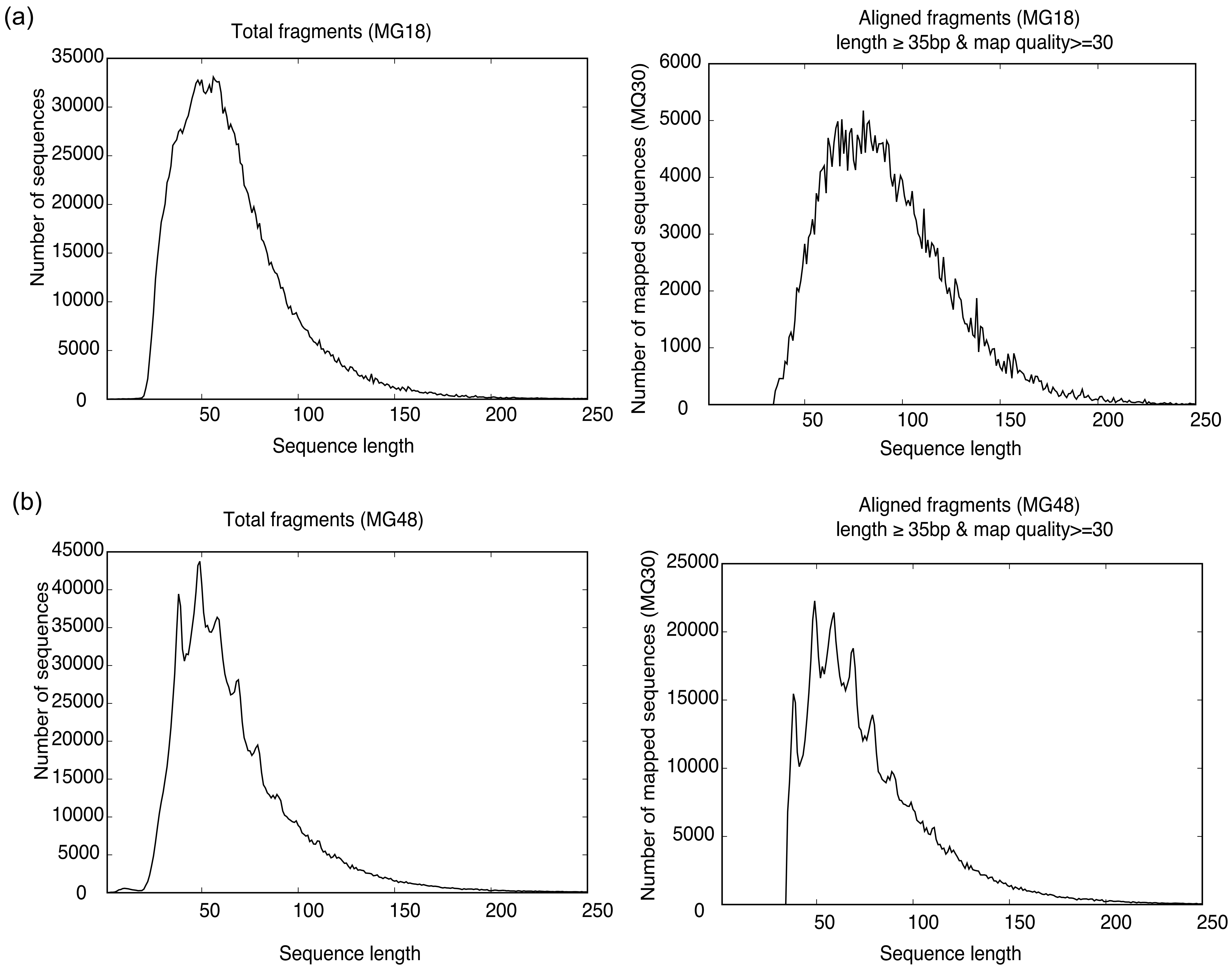

(a)
Total fragments (MG18)
Number of sequences
Sequence length
Aligned fragments (MG18)
length ≥ 35bp & map quality>=30
Number of mapped sequences (MQ30)
Sequence length
(b)
Total fragments (MG48)
Number of sequences
Sequence length
Aligned fragments (MG48)
length ≥ 35bp & map quality>=30
Number of mapped sequences (MQ30)
Sequence length
